# Protocol for co-culture of murine bone marrow-derived dendritic cells with OT-I mouse CD8^+^ T cells to evaluate immune activation efficacy

**DOI:** 10.1016/j.xpro.2026.104621

**Published:** 2026-06-10

**Authors:** Min Jiang, Dana Matzek, Anny Nguyen, Bastian Popper, Olivia M. Merkel

**Affiliations:** 1 Ludwig-Maximilians-Universität München, Department of Pharmacy, Butenandtstraße 5, 81377 Munich, Germany; 2 Member of the German Center for Lung Research (DZL), Munich, Germany; 3 Ludwig-Maximilians-Universität München, Biomedical Center, Großhaderner Str. 9, 82152 Planegg- Martinsried, Germany; 4 Ludwig-Maximilians-Universität München, Core Facility Animal Models, Großhaderner Str. 9, 82152 Planegg- Martinsried, Germany; 5 Center for Nanoscience (CeNS) Munich, Munich, Germany; 6 Cluster for Nucleic Acid Therapeutics Munich (CNATM), Munich, Germany

**Keywords:** Cell-based Assays, Health Sciences, Immunology, Model Organisms

## Abstract

Preliminary evaluation of vaccine-induced immune activation can be performed with minimal animal use, in line with the principles of the 3Rs (replacement, reduction, and refinement) in animal experimentation. Here, we present a protocol to assess murine antigen-specific CD8^+^ T cell activation using a co-culture model of bone marrow-derived dendritic cells (BMDCs) and OT-I transgenic CD8^+^ T cells. We describe the steps for transfecting BMDCs with antigen-encoding mRNA formulations and assessing T cell responses after co-culture by intracellular cytokine staining and proliferation.

For complete details on the use and execution of this protocol, please refer to Jiang et al.^1^

## Highlights

• Steps for isolating bone marrow using centrifugation-based approach
• Protocol for generating BMDCs from murine bone marrow
• Instructions for isolating OT-I CD8+ T cell by magnetic separation
• Guidance on co-culture and functional readouts of CD8^+^ T cell activation

## Abstract (editor highlights)

Publisher’s note: Undertaking any experimental protocol requires adherence to local institutional guidelines for laboratory safety and ethics.

## Graphical abstract

**Figure:**
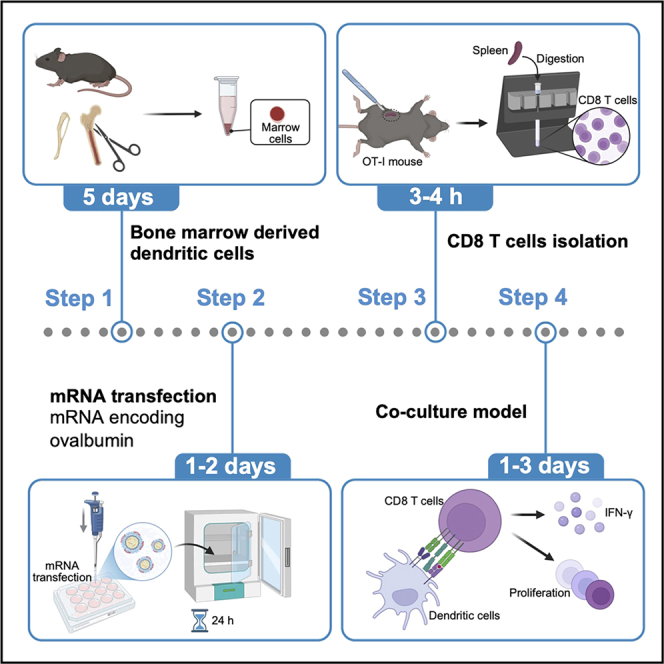

## Before you begin

Evaluation of vaccine-induced immune activation is a central determinant in the development and optimization of emerging vaccine platforms. However, direct assessment of antigen-specific immune responses often relies on extensive *in vivo* experimentation, which limits throughput and flexibility during early-stage evaluation. *Ex vivo* systems that recapitulate key steps of antigen presentation and T cell activation therefore provide an accessible alternative for preliminary screening of vaccine candidates.

We previously established a co-culture system using bone marrow-derived dendritic cells (BMDCs) from C57BL/6J mice and CD8^+^ T cells isolated from OT-I transgenic mice (C57BL/6-Tg(TcraTcrb)1100Mjb/J). OT-I mice carry transgenes encoding explicit TCR α (Tcra-V2) and TCR β (Tcrb-V5) chains, generating CD8^+^ T cells that specifically recognize the OVA257-264 peptide (SIINFEKL) presented by the MHC-I allogen H-2Kb.^2^ Following transfection of BMDCs with ovalbumin (OVA)-encoding mRNA formulations, we assess antigen presentation-driven CD8^+^ T cell activation using intracellular cytokine staining (ICS) and T cell proliferation as functional readouts. This assay also demonstrates a high degree of antigen specificity, because CD8^+^ T cells are not activated when BMDCs receive the same carrier loaded with mRNA encoding an irrelevant antigen, such as the SARS-CoV-2 spike protein.^1^

In this protocol, we use mRNA-based vaccine formulations as a representative example, however, this workflow enables the evaluation of other antigen formats or delivery systems with appropriate optimization. Regardless of the antigen format, including DNA, mRNA, circular RNA, protein, or peptide, this protocol evaluates responses only in the OVA antigen context, specifically the H-2Kb-presented OVA257-264 epitope SIINFEKL, due to the use of OT-I transgenic CD8^+^ T cells.

### Innovation

This protocol presents a streamlined and reproducible workflow for the preliminary evaluation of vaccine-induced CD8^+^ T cell activation using an *ex vivo* co-culture system in line with the principles of the 3Rs (Replacement, Reduction and Refinement) in animal experimentation. It provides a systematic and functionally integrated approach that links dendritic cell-mediated antigen presentation with downstream CD8^+^ T cell activation within a single experimental framework. The use of OT-I transgenic mice enables sensitive and standardized detection of antigen-specific responses, facilitating precise comparison of vaccine functionality across different formulations or delivery platforms. In contrast to approaches that rely solely on quantifying surface H-2Kb-SIINFEKL complexes as a measure of antigen presentation, or on assessing costimulatory markers such as CD40, B7-1/2, and MHC-II expression, this cascade-based functional assay integrates both antigen presentation and costimulatory signaling through downstream CD8^+^ T cell activation. By reflecting the combined effect of these signals, the system provides enhanced sensitivity and functional resolution, allowing the assessment of subtle differences in immune activation between vaccine formulations.

In addition, this protocol incorporates an optimized bone marrow cell isolation step that replaces conventional marrow flushing with a rapid centrifugation-based approach. This alternative method minimizes prolonged handling of bones, reduces liquid splashing associated with repeated flushing, and lowers the risk of contamination, thereby improving operational robustness and experimental consistency.

### Institutional permissions

Experiments involving mice must conform to institutional and governmental guidelines. The Government of Upper Bavaria, Munich, Germany approved all animal procedures in this protocol (5.1–231 5682/LMU/BMC/CAM). The animal experiments also complied with European Directive 2010/63/EU and the German law of animal protection (TSchG).

### Preparation of RPMI-1640 complete culture medium

**Timing: 15 min**

1. Add supplements required for complete RPMI-1640 culture medium.

**Table:** 

Reagent	Final concentration	Amount
RPMI-1640 medium	N/A	445 mL
Fetal bovine serum	10% (v/v)	50 mL
Penicillin-streptomycin 10,000 U/mL	1% (v/v)	5 mL

***Note:*** Store the complete medium at 4°C and warm to 37°C before use. RPMI-1640 complete medium serves as the basal medium for subsequent preparation of DC medium and for co-culture of BMDCs with CD8^+^ T cells.

### Preparation of DC medium

**Timing: 30 min**

2. Add supplements required for cell culture medium of dendritic cells.

**Table:** 

Reagent	Final concentration	Amount
RPMI-1640 complete culture medium	N/A	495 mL
HEPES buffer	10 mM	5 mL
β-Mercaptoethanol	50 μM	1.75 μL

***Optional:*** The volume of β-Mercaptoethanol required from the original 14.3 M stock is very small (1.75 μL per 500 mL medium) and may be difficult to pipette accurately, especially when preparing smaller batches. To improve accuracy, pre-dilute the stock solution 1:100 in sterile PBS to 0.143 M. In this case, add 175 μL of diluted solution to achieve a final concentration of 50 μM. Prepare DC culture medium in smaller batches and use it within 4 weeks after preparation.

***Note:*** Store DC culture medium at 4 °C for up to 4 weeks.

**CRITICAL:** To enable efficient differentiation of bone marrow cells into dendritic cells, add mouse Granulocyte-macrophage colony-stimulating factor (GM-CSF) recombinant protein freshly to a final concentration of 20 ng/mL before use.

### Preparation of FACS buffer

**Timing: 30 min**

3. Prepare 0.2 M EDTA-2Na stock solution and filter it using a 0.22 μm filter.

**Table:** 

Reagent	Final concentration	Amount
EDTA-2Na	0.2 M	3.72 g
Double-distilled water (ddH_2_O)	N/A	To 50 mL
  a. Dissolve 3.72 g EDTA-2Na in approximately 35–40 mL of ddH_2_O with continuous stirring.
  b. Adjust the pH to 8.0 ± 0.1 using 10 M NaOH to facilitate complete dissolution of EDTA.
  c. Add the final volume to the corresponding volume of ddH_2_O to obtain a total of 50 mL.
  d. Sterilize the solution by passing it through a 0.22 μm filter.
    ***Note:*** The EDTA-2Na stock solution can be stored at 4 °C for long-term use.
4. Add supplements required for FACS buffer.

**Table:** 

Reagent	Final concentration	Amount
1x PBS without Ca^2+^/Mg^2+^	N/A	97 mL
Fetal bovine serum	2% (v/v)	2 mL
0.2 M EDTA-2Na stock solution	2 mM	1 mL

### Preparation of MACS buffer

**Timing: 30 min**

5. Prepare 0.2 M EDTA-2Na stock solution as described above in step 3.
6. Add supplements required for MACS buffer.

**Table:** 

Reagent	Final concentration	Amount
1x PBS without Ca^2+^/Mg^2+^	N/A	94 mL
MACS® BSA Stock Solution	0.5% (w/v)	5 mL
0.2 M EDTA-2Na stock solution	2 mM	1 mL

## Key resources table

**Table:** 

REAGENT or RESOURCE	SOURCE	IDENTIFIER
**Experimental models: Organisms/strains**		

C57BL/6J mice (6–8 weeks, female and male)	The Jackson Laboratory	Stock no. 000664; RRID: IMSR_JAX:000664
OT-I transgenic mice (C57BL/6-Tg (TcraTcrb)1100Mjb/J)	The Jackson Laboratory	Stock no. 003831; RRID: IMSR_JAX:003831

**Antibodies**		

CD8a FITC clone 53–6.7 (Dilution: 2 μL in 100 μL FACS buffer)	Miltenyi Biotec	Cat. no. 130-118-468
CD11c PE clone N418 (Dilution: 1.25 μL in 100 μL FACS buffer)	BioLegend	Cat. no. 117308
IFN-γ APC clone XMG1.2 (Dilution: 2.5 μL in 100 μL FACS buffer)	BioLegend	Cat. no. 505810
CD8a APC-Cyanine7 clone 53–6.7 (Dilution: 2.5 μL in 100 μL FACS buffer)	BioLegend	Cat. no. 100713
TruStain FcX™ (anti-mouse CD16/32) Antibody (Dilution: 2 μL in 100 μL FACS buffer)	BioLegend	Cat. no. 101320

**Chemicals, peptides, and recombinant proteins**		

RPMI-1640 medium	Merck (Sigma-Aldrich)	Cat. no. R8758
Dulbecco′s Phosphate Buffered Saline	Merck (Sigma-Aldrich)	Cat. no. D8573
Molecular biology grade ethanol	Merck (Sigma-Aldrich)	Cat. no. E7023
β-Mercaptoethanol	Merck (Sigma-Aldrich)	Cat. no. M6250
Penicillin-Streptomycin	Merck (Sigma-Aldrich)	Cat. no. P4333
Ethylenediaminetetraacetic acid disodium salt dihydrate (EDTA-2Na)	Merck (Sigma-Aldrich)	Cat. no. E4884
Zombie Violet™ Fixable Viability Kit (Dilution: 1 μL in 100 μL PBS)	BioLegend	Cat. no. 423113
CellTrace™ CFSE Cell Proliferation Kit	BioLegend	Cat. no. 423801
Monensin Solution (1,000×)	BioLegend	Cat. no. 420701
Fetal bovine serum	Thermo Fisher Scientific	Cat. no. 26140-079
Gibco™ HEPES buffer (1M)	Thermo Fisher Scientific	Cat. no. 15630080
Mouse GM-CSF Recombinant Protein, PeproTech®	Thermo Fisher Scientific	Cat. no. 315-03-20UG
Lipofectamine™ 2000 Transfection Reagent	Thermo Fisher Scientific	Cat. no. 11668019
Paraformaldehyd, 4 % in PBS	Thermo Fisher Scientific	Cat. no. J61899.AP
CD8a^+^ T Cell Isolation Kit, mouse	Miltenyi Biotec	Cat. no. 130-104-075
MACS® BSA Stock Solution	Miltenyi Biotec	Cat. no. 130-091-376
Red Blood Cell Lysis Solution (10×)	Miltenyi Biotec	Cat. no. 130-094-183

**Oligonucleotides**		

Ovalbumin mRNA (Cap1, m1Ψ)	Genescript	Cat. no. RP-A00043

**Critical commercial assays**		

eBioscience™ Foxp3/Transcription Factor Staining Buffer Set	eBioscience (Thermo Fisher)	Cat. no. 00-5523-00

**Software and algorithms**		

FlowJo v10	BD Bioscience	RRID: SCR_008520

**Other**		

70 μm cell strainer	Corning	Cat. no. 431751
LS columns	Miltenyi Biotec	Cat. no. 130-042-401
MidiMACS™ separator	Miltenyi Biotec	Cat. no. 130-042-302
Falcon® 100 mm x 15 mm Not TC-treated Bacteriological Petri Dish, Sterile	CORNING	Cat. no. 351029
12-well plate for cell culture	Greiner Bio-One	Cat. no. 665180
6-well plate for cell culture	Greiner Bio-One	Cat. no. 657160
1.5 mL microcentrifuge tubes	Greiner Bio-One	Cat. no. 616201
20-gauge needle (Sterican®)	B. Braun	Cat. no. 281C.1
PCR tubes (0.2 mL)	Starlab	Cat. no. I1402-3700
Attune™ NxT Flow Cytometer (405/488/637 nm configuration)	Thermo Fisher Scientific	Model: AFC2
CASY® Cell Counter	OMNI Life Science	CASY TTT

## Step-by-step method details

### Isolation of bone marrow cells from C57BL/6J mice

**Timing: 3 h**

This section describes the isolation of bone marrow cells from murine femurs and tibiae and the corresponding preparation for differentiation into BMDCs.

***Note:*** We recommend using C57BL/6J mice aged 6-8 weeks. Either male or female mice are suitable for this protocol.

1. Harvest femurs and tibiae.
  a. Sacrifice a C57BL/6J mouse by cervical dislocation according to institutional animal care guidelines.
  b. Place the mouse in a prone position on a dissecting board and spray the hind legs and lower back thoroughly with 70% (v/v) ethanol.
  c. Incise the dorsal skin and cut through the hip joint to detach the entire hind limbs.
  d. Remove the skin completely and expose the bones.
  e. Gently remove muscles and adipose tissue from the femurs and tibiae using scissors and tweezers. If needed, carefully rub off residual tissue using gloved fingers.
  f. Separate the femur from the tibia by cutting through the knee joint.^3^
  g. Cut off the paws distal to the ankle and remove any remaining skin and muscle.
  h. Transfer femurs and tibiae harvested from one single mouse into a 50 mL Falcon tube containing ice-cold sterile PBS and keep on ice until further processing.

**CRITICAL:** Remove as much soft tissue as possible in this step without damaging or fracturing the bones.

***Note:*** Do not pool the bones harvested from different mice.

2. Disinfect and wash the bones.
  a. Prepare a 6-well plate inside a biosafety cabinet as follows:
    i. 1 well containing 3 mL of 70% (v/v) ethanol.
    ii. 3 wells containing 3 mL of DC medium.
  b. Transfer the bones into the ethanol-containing well and soak for 3 min for surface disinfection.
  c. Sequentially transfer the bones into the three wells filled with DC medium for a quick dip-and-lift to wash off residual ethanol.

***Note:*** After ethanol treatment, it is easy to remove remaining soft tissues. Use sterile scissors and tweezers to gently clean the bones if necessary.

**Figure 1:**
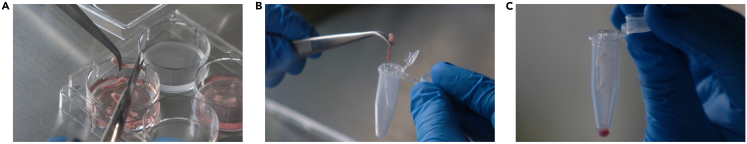
Harvest of bone marrow using a centrifugation-based method (A) One end of each femur and tibia is cut to expose the marrow cavity. (B) The bone is placed vertically into a perforated PCR tube nested within a 1.5 mL microcentrifuge tube. (C) Bone marrow cells are collected by brief centrifugation, resulting in extrusion of marrow into the collection tube.
  c. Place one bone per PCR tube with the open end facing downward and close the attached lid (Figure 1B).
  d. Nest each PCR tube into a 1.5 mL microcentrifuge tube.
  e. Centrifuge at 10,000 × *g* for up to 15 s to spin the bone marrow into the collection tube.
  f. Inspect visually: the bones should appear white, and a visible pellet should be present in the microcentrifuge tube (Figure 1C).

***Note:*** Red blood cell (RBC) lysis is not necessary at this step. In our experience, omitting RBC lysis better preserves cell viability and results in a higher BMDC yield, without compromising the purity of differentiated BMDCs.

3. Collect the bone marrow cells.
  a. Use a 20-gauge needle to puncture a small hole at the bottom of each PCR tube. Prepare four such PCR tubes for the bones collected from one mouse.
  b. Cut one end of each femur and tibia to expose the marrow cavity (Figure 1A).

4. Resuspend each marrow pellet in 1 mL fresh DC medium.
5. Pool suspensions and filter through a 70 μm cell strainer into a new 50 mL Falcon tube to remove any remaining bone or muscle fragments.
6. Centrifuge at 200 × *g* for 10 min and resuspend the pellet in 2 mL DC medium.
7. Count cells using a cell counter, e.g., CASY counter (OMNI Life Science, Bremen, Germany).
8. Adjust the concentration to 1×10^7^ cells/mL.
9. Seed 1×10^7^ cells in a 100 mm bacteriological petri dish with a total volume of 10 mL DC medium containing 20 ng/mL mouse GM-CSF.
10. Record this day as day 0 of differentiation.

**CRITICAL:** Use bacteriological petri dishes to culture bone marrow cells.^4^ As they are non tissue-culture (TC)-treated dishes, their use can minimize macrophage adherence and overgrowth.

***Note:*** Bone marrow harvested from one C57BL/6J mouse typically yields 4–7×10^7^ cells.

### Differentiation and collection of BMDCs

**Timing: 5 days**

This section describes the differentiation of bone marrow cells into BMDCs and their collection for downstream assays.

11. On day 3, gently add 10 mL pre-warmed DC medium supplemented with freshly added 20 ng/mL mouse GM-CSF to each dish.

**Figure 2:**
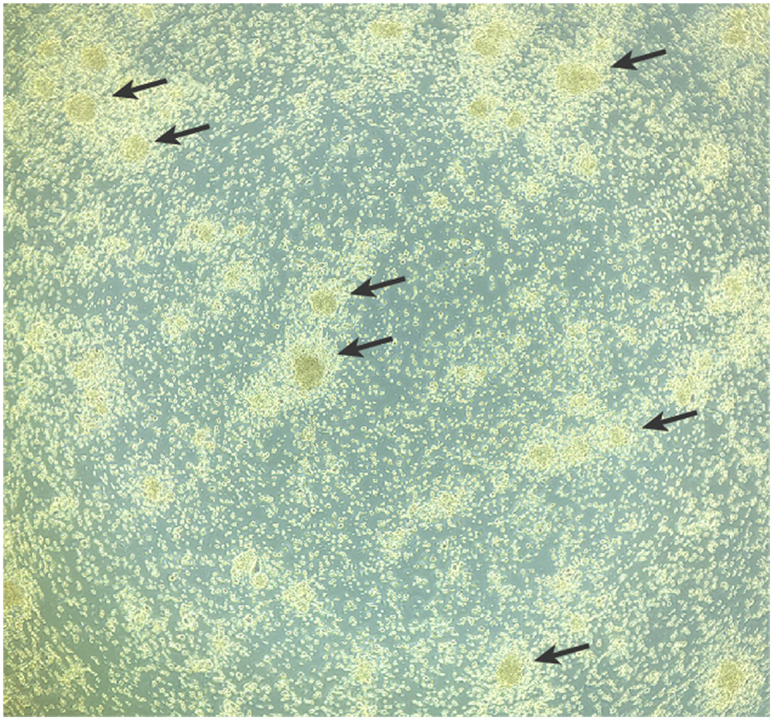
Bone marrow-derived dendritic cells (BMDCs) form loosely adherent colonies during differentiation Representative bright-field image showing BMDC colonies on day 3 of culture (black arrows indicate cell clusters). Image was captured as a qualitative overview and calibrated scale information is not available.

**CRITICAL:** On day 3, confirm the formation of loosely adherent cell clusters in BMDC cultures under the microscope (Figure 2). These clusters are highly sensitive to mechanical disturbance. Handle dishes gently and add medium slowly along the dish wall.

**Figure 3:**
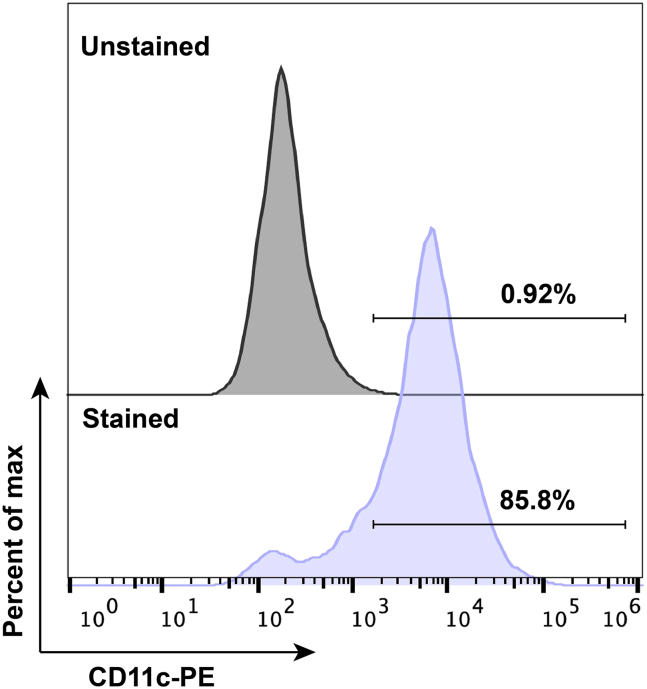
Purity of bone marrow-derived dendritic cells (BMDCs) after differentiation Representative flow cytometry analysis showing the percentage of CD11c^+^ cells in the collected BMDCs.

***Note:*** BMDCs are suitable for downstream experiments when the detected percentage of CD11c^+^ reaches at least 80%.

12. Collection of BMDCs.
  a. On day 5, transfer petri dishes carefully to a biosafety cabinet.
  b. Gently decant the supernatant. Alternatively, aspirate the supernatant carefully using a vacuum pump or a serological pipette, while avoiding disturbance of the loosely adherent BMDC clusters.
  c. Add 3 to 4 mL fresh DC medium and gently pipette 2 to 3 times to detach loosely adherent BMDC clusters.
    **CRITICAL:** Excessive pipetting force may detach adherent macrophages and reduce BMDC purity.
  d. Collect cell suspension in a 50 mL Falcon tube and centrifuge at 200 × *g* for 10 min.
  e. Resuspend the cell pellet in 2 mL of DC medium and count cells using a cell counter (e.g., Casy, OMNI Life Science).
    ***Note:*** In our experience, the viability of collected BMDCs at this stage is typically above 80%, and one mouse yields approximately 1–3×10^7^ cells on day 5 under our culture conditions. However, the final yield may vary depending on mouse age, reagent quality and handling during differentiation.
13. Assessment of BMDCs purity.
  a. Aliquot 1×10^6^ cells into two 1.5 mL tubes and centrifuge at 400 x g for 5 min.
  b. Resuspend one pellet in 100 μL prepared FACS buffer (unstained control).
  c. Resuspend the other pellet in 50 μL diluted anti-mouse CD16/CD32 (Fc block), then add 50 μL diluted PE-conjugated anti-mouse CD11c antibody and incubate at 4°C for 30 min.
  d. Wash twice with 1 mL FACS buffer and resuspend in 500 μL FACS buffer.
  e. Check the purity of collected dendritic cells by evaluating the percentage of the PE-positive population (Figure 3) using a Flow cytometer (e.g., Attune N×T, Thermo Fisher Scientific).

### Transfection of BMDCs

**Timing: 1–2 days**

This section describes antigen loading of BMDCs and is particularly applicable for evaluating carrier systems developed for vaccine delivery. In this protocol, we use mRNA-based vaccination as a representative example.

14. Prepare pre-warmed DC-medium supplemented with freshly added mouse GM-CSF at a final concentration of 20 ng/mL.

***Optional:*** Mouse GM-CSF is not strictly required at this step. However, we find that supplementation with GM-CSF improves BMDCs viability and overall cell health.

15. Seed BMDCs at a density of 6×10^5^ cells per well in 12-well plates and culture them in 1 mL DC medium containing mouse GM-CSF.
16. After 12–18h of incubation, treat each well with formulations containing 1 μg ovalbumin (OVA) mRNA and incubate for 24 h.
  ***Note:*** The complete OVA mRNA sequence information is available from the source link provided in the Key Resources Table.
  a. For polymeric formulations evaluated in this protocol, including PBAE and PLGA/PBAE formulations, prepare nanoparticles at a polymer:mRNA weight ratio of 60:1 by mixing 60 μL of the polymer phase with 30 μL of mRNA solution containing 1 μg mRNA. Apply the resulting 90 μL formulation to each well.^1^
  b. For mRNA vaccine studies, include a commercial Lipofectamine reagents as a positive control. This protocol uses Lipofectamine 2000 at 1 μL per 1 μg mRNA, within the manufacturer’s recommended range. Add the same final formulation volume of 90 μL to each well.
    ***Note:*** Formulations without any mRNA serve as controls for assessing the non-antigen-specific immune activation induced by the carrier system.
    ***Note:*** Formulations loaded with mRNA encoding an irrelevant antigen serve as controls for antigen specificity.

### Isolation of CD8^+^ T cells from OT-I mice

**Timing: 3–4 h**

This section describes the isolation of antigen-specific CD8^+^ T cells from spleens of OT-I transgenic mice.

**CRITICAL:** Initiate CD8^+^ T cell isolation at least 3 h before completion of BMDCs transfection to ensure synchronized co-culture.

17. Harvest spleens from OT-I mice.
  ***Note:*** Use adult OT-I mice aged 12–24 weeks for this protocol. Previous studies showed age-related alterations in OT-I CD8^+^ T cell phenotype and function in elderly mice aged 70-100 weeks.^5^ Therefore, keep donor age consistent within each experiment. In our experience, sex matching between OT-I T cell donors and BMDC donors is not required. However, record donor sex and preferably keep it consistent within each experiment.
  a. Sacrifice C57BL/6-Tg (TcraTcrb)1100Mjb/J (OT-I) mice by cervical dislocation in accordance with institutional animal care guidelines.
  b. Place the mouse in a prone position on a dissecting platform and disinfect the left dorsal side with 70% (v/v) ethanol.
  c. Carefully incise the skin and underlying tissue to expose and harvest the spleen.
    ***Note:*** OT-I mice exhibit a high frequency of CD8^+^ T cells compared with wild type C57BL/6 mice.^2^ Typically, each adult spleen yields 0.5–2.5×10^7^ CD8^+^ T cells can be obtained per adult spleen. In our experience, around 7×10^7^ CD8^+^ T cells are isolated from four adult spleens.
  d. Place harvested spleens into sterile PBS or RPMI-1640 complete culture medium and transfer them to a biosafety cabinet. Perform subsequent steps under sterile conditions.
18. Preparation of single cell splenocyte suspension.
  a. Add 3 mL RPMI-1640 complete culture medium to each well of a 6-well plate and place a 70 μm cell strainer into each well.
  b. Gently grind each spleen through the screener using the plunger end of a 1 mL syringe and collect the cell suspension in a 15 mL Falcon tube.
    i. Use one cell strainer per spleen.
    ii. Reuse the syringe plunger if processing multiple spleens.
    iii. Rinse each strainer with an additional 1 mL RPMI-1640 medium after dissociation.
    iv. Pool cell suspension from up to two spleens into one 15 mL Falcon tube.
  c. Centrifuge the cell suspension at 300 × *g* for 10 min and discard the supernatant.
19. Red blood cell lysis.
  a. Prepare 1× red blood cell (RBC) lysis solution (Miltenyi) by 1:10 diluting the 10× stock with ddH_2_O and store at 20–25°C.
  b. Resuspend the cell pellet (from up to two spleens) in 1 mL RPMI-1640 medium.
  c. Add 10 mL 1× RBC lysis buffer, vortex briefly for 5 s, and incubate for 2 min at 20–25°C.
  d. Centrifuge at 300 × *g* for 10 min and aspirate the supernatant.
  e. Resuspend the cell pellet in 2 mL medium and determine cell concentration using a cell counter (e.g., Casy counter).

***Note:*** One adult spleen typically yields 2–3×10^8^ splenocytes after red blood cells lysis under our experimental conditions.

20. Perform manual magnetic labeling using mouse CD8a^+^ T Cell Isolation Kit (Miltenyi) according to the manufacturer’s instructions.

***Note:*** The isolation strategy uses negative selection, in which magnetic labeling targets non-CD8a^+^ T cells.

Use a minimum volume of 500 μL for subsequent magnetic separation. One adult spleen typically yields 2–3×10^8^ total cells, which is sufficient to meet this requirement.

**CRITICAL:** Work fast, keep cells cold and use pre-cooled solutions (2–8°C).

21. Manual magnetic cell separation.
  a. Perform the separation in a biosafety cabinet. Disinfect all materials before use.
  b. Place an LS column into the magnetic field of a suitable magnetic separator (e.g., MACS, Miltenyi). Here, we use the MidiMACS separator.
    i. Maximal labeled cells per column: ∼1 × 10^8^.
    ii. Maximal total cells per column: 2 × 10^9^.
  c. Rinse the column with 3 mL MACS buffer.
  d. Apply cell suspension onto the LS column and collect the flow-through containing unlabeled CD8^+^ T cells.
  e. Wash the column with 3 mL of MACS buffer and collect the flow-through. Combine it with the fraction collected in step 21.d.

**CRITICAL:** Apply MACS buffer or cell suspension in 1 mL increments, not all at once. Clearly label collection tubes for waste and isolated cells to avoid cross-contamination.

**Figure 4:**
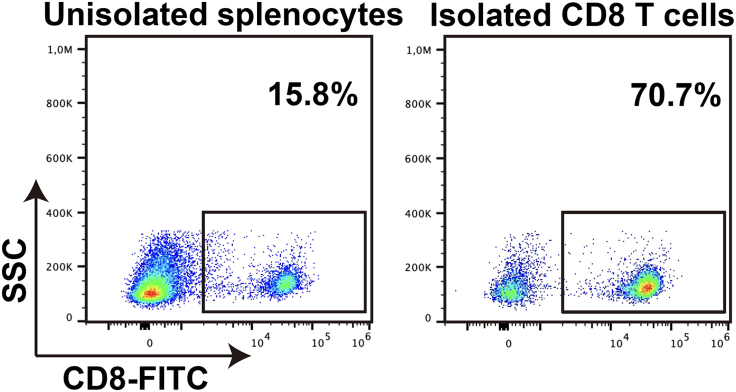
Purity of CD8^+^ T cells after magnetic isolation Representative flow cytometry analysis showing the percentage of CD8^+^ T cells following negative selection.

22. Count and seeding of CD8^+^ T cells.
  a. Count the cells using a cell counter.
  b. Adjust the cell concentration to 1×10^6^ cells/mL using RPMI-1640 complete culture medium.
  c. Seed 1×10^6^ CD8^+^ T cells per well in 6-well plates for subsequent co-culture experiments.
23. CFSE labeling of CD8^+^ T cells (optional).
  ***Note:*** For optional proliferation assays, label CD8^+^ T cells with CFSE before seeding them for co-culture with transfected BMDCs.
  a. Perform CFSE labeling of CD8^+^ T cells according to the manufacturer’s instructions.
  b. In this protocol, centrifuge 2.5 × 10^7^ CD8^+^ T cells at 400 × *g* for 5 min.
  c. Resuspend the pellet in 2 mL CFSE solution (4 μM diluted in PBS) and incubate for 15 min at 20–25°C, protected from light.
  d. Add 12 mL RPMI-1640 complete medium to quench staining and centrifuge to pellet the cells.
  e. Resuspend CFSE-stained CD8^+^ T cells and use them directly for co-culture with transfected BMDCs.
24. (Optional) Purity assessment of CD8^+^ T cells.
  ***Note:*** Perform this step after setting up the co-culture if needed. Because antibody staining and flow cytometric analysis take time, seeding the cells first may help maintain optimal cell conditions.
  a. Aliquot 1×10^6^ cells into two 1.5 mL microcentrifuge tubes and centrifuge at 400 × *g* for 5 min.
  b. Resuspend one pellet in 100 μL of FACS buffer (unstained control).
  c. Resuspend the other pellet in 50 μL diluted anti-Mouse CD16/CD32, then add 50 μL diluted FITC-conjugated anti-mouse CD8 antibody and incubate for 30 min at 4°C.
  d. Add 1 mL FACS buffer to stop staining and centrifuge at 400 x g for 5 min.
  e. Wash once more with FACS buffer and resuspend the pellet in 500 μL FACS buffer.
  f. Analyze CD8^+^ T cell purity by flow cytometry (Attune NxT) based on the percentage of FITC-positive population (Figure 4).

### Co-culture of transfected BMDCs and CD8^+^ T cells

**Timing: 1–3 days**

This section describes the co-culture of antigen-loaded BMDCs with CD8^+^ T cells and provides a recommended cell ratio for functional immune activation assays.

25. At 24 h post-transfection, collect BMDCs from plates and transfer them into sterile 1.5 mL microcentrifuge tubes respectively.
26. Aliquot 100 μL of the BMDCs suspension from each tube and count cells.
27. Centrifuge the remaining cells at 400 × *g* for 5 min to pellet the BMDCs.
28. Resuspend the cell pellet in fresh RPMI-1640 medium and adjust the concentration to 2.5×10^5^ cells/mL.
29. Add 800 μL of the BMDC suspension to each well of 6-well plates containing 1×10^6^ CD8^+^ T cells and label the wells accordingly.

***Note:*** The BMDC:CD8^+^ T cell ratio used in this protocol is 1:4. Published DC-T cell co-culture assays have used different ratios, commonly ranging from 1:2 to 1:10, depending on the experimental setup.^6^^,^^7^ The optimal ratio may therefore be variable based on the delivery system, antigen format and intended assay readouts. Keep the ratio consistent within the same experiment to allow reliable comparison between groups.

30. Put the plates back to the incubator and co-culture under the following conditions as required for the indicated assays:
  a. 6 h for intracellular cytokine staining (ICS).
    i. After 1 h of co-culture, add 200 μL of 10× monensin solution (prepared from a 1000× stock, diluted in RPMI-1640 complete medium) to achieve a final concentration of 1× monensin and a total volume of 2 mL per well.
  b. 72 h for CFSE-based proliferation assays.
    i. Add 200 μL fresh RPMI-1640 complete medium to adjust the total volume to 2 mL per well.

***Note:*** Perform ICS and CFSE assays independently. Prepare separate co-culture plates in parallel for each assay.

### Flow cytometric assessment of CD8^+^ T cell activation

**Timing: 6–8 h**

**Figure 5:**
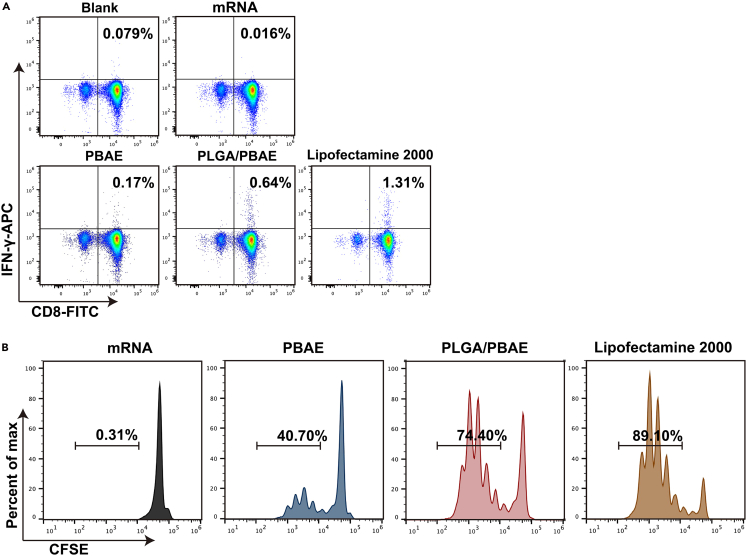
Antigen-specific CD8^+^ T cell activation after co-culture with transfected BMDCs (A) Percentages of IFN-*γ*-producing CD8^+^ T cells after 6 h of co-culture with transfected BMDCs. (B) CD8^+^ T cell proliferation assessed by carboxyfluorescein succinimidyl ester (CFSE) dilution after 72 h of co-culture, shown as the percentage of divided cell subsets.
32. CFSE assay readout.
  a. After 72 h of co-culture, collect cells and centrifuge at 400 x g for 5 min.
  b. Wash cell pellets once with PBS and resuspend in 100 μL of Zombie Violet viability dye (1:100 dilution in PBS) and incubate at 20–25°C for 20 min.
  c. Add 1 mL of FACS buffer to stop staining and centrifuge at 400 × *g* for 5 min.
  d. Resuspend the cell pellet in 50 μL FACS buffer containing anti-mouse CD16/CD32 (Fc block).
  e. Add another 50 μL FACS buffer containing APC-Cyanine7-conjugated anti-mouse CD8 antibody and incubate at 4°C for 30 min.
  f. Wash twice with FACS buffer and resuspend in 300–500 μL of FACS buffer for flow cytometric analysis (Figure 5B).

***Note:*** Acquire samples by flow cytometry immediately whenever possible. If immediate acquisition is not possible, fix samples with 4% paraformaldehyde (PFA) or another appropriate fixation reagent compatible with the viability dye, CFSE and fluorophore-conjugated antibodies. Store fixed samples at 4 °C protected from light and acquire as soon as possible to preserve fluorescence signals.

31. Intracellular cytokine staining.
  a. After 6 h of co-culture, collect cells from each well and centrifuge at 400 × *g* for 5 min at 20-25°C.
  b. Wash once with PBS and centrifuge again at 400 × *g* for 5 min.
  c. Resuspend the pellet in 100 μL of Zombie Violet viability dye (1:100 dilution in PBS) and incubate at 20–25°C for 20 min.
  d. Add 1 mL of FACS buffer to stop staining and centrifuge at 400 × *g* for 5 min.
  e. Resuspend the cell pellet in 50 μL FACS buffer containing anti-mouse CD16/CD32 (Fc block).
  f. Add another 50 μL FACS buffer containing FITC-conjugated anti-mouse CD8 antibody and incubate at 4°C for 30 min.
  g. Add 1 mL FACS buffer, centrifuge, and proceed with fixation and permeabilization using the eBioscience™ Foxp3/Transcription Factor Staining Buffer Set according to the manufacturer’s instructions.
  h. Stain cells with APC-conjugated anti-mouse IFN-γ antibody in permeabilization buffer for 30 min at 20–25°C.
  i. Wash twice with FACS buffer and resuspend in 300–500 μL of FACS buffer for flow cytometric analysis (Figure 5A).

## Expected outcomes

Antigen-loaded BMDCs after OVA mRNA transfection can induce robust antigen-specific activation of CD8^+^ T cells isolated from OT-I mice, as shown by increased cytokine secretion and T cell proliferation (Figure 5). Control conditions lacking antigen or containing irrelevant antigen show minimal activation.^1^

## Quantification and statistical analysis

Analyze flow cytometry data using FlowJo v10. Use unstained and single-stained controls for gate setting and compensation. For intracellular cytokine staining, gate viable CD8^+^ T cells by excluding Zombie Violet-positive dead cells and selecting CD8^+^ populations. Quantify IFN-γ-producing cells as the percentage of IFN-γ^+^ cells among viable CD8^+^ T cells. For CFSE-based proliferation assays, gate viable CD8^+^ T cells using the same strategy and quantify proliferation based on CFSE dilution as the percentage of divided cells. Apply the same gating strategy consistently to all samples within one experiment. Export data as Excel files and analyze them using GraphPad Prism or equivalent statistical software.

## Limitations

This protocol enables preliminary evaluation of vaccine-induced T cell activation using an *ex vivo* co-culture model, but it has several inherent limitations. First, the assay relies on CD8^+^ T cells isolated from OT-I transgenic mice, which restricts antigen specificity to the ovalbumin context. Second, as an *ex vivo* system, it cannot fully recapitulate the complexity of the *in vivo* immune environment. Factors such as antigen distribution, lymphatic drainage dynamics, and the contributions of diverse immune cell subsets are not captured in this model. Therefore, interpret results from this workflow as indicators of early-stage immune activation rather than as direct predictors of *in vivo* immunogenicity. In addition, the efficiency of dendritic cell differentiation and maturation may vary depending on mouse age, bone marrow quality, used reagents and handling during the whole procedure. Careful standardization of bone marrow isolation and culture conditions is therefore essential for reproducible results.

## Troubleshooting

### Problem 1

The bone marrow cavity remains red after centrifugation (step 3), or bone marrow remains trapped either at the bone ends or the bottom of PCR tube.

### Potential solution

If the bone cavity remains red, re-cut one end of the bone to fully expose the marrow cavity and repeat centrifugation.

If bone marrow remains at the bone end, flush the bone gently with 1 mL DC medium using a 1 mL syringe fitted with a 27-gauge needle to remove residual marrow, and if necessary, repeat the flushing step.

If bone marrow cells remain trapped at the bottom of the PCR tube, gently resuspend them with approximately 100 μL DC medium using a 200 μL pipette. Pipette up and down 4 to 5 times, and transfer the recovered cell suspension into the corresponding collection tube.

### Problem 2

Bone marrow cells fail to differentiate into visible dendritic cell colonies (step 11).

### Potential solution

Poor differentiation may result from suboptimal reagent quality or improper culture handling based on some previous experiences.^8^

First, verify the quality of used fetal bovine serum (FBS). Avoid using FBS that has undergone multiple freeze-thaw cycles (>2 cycles) and confirm that other cell lines grow normally in medium prepared with the same FBS. Ensure that the serum has been properly heat-inactivated if required.

Second, evaluate the quality of used murine GM-CSF recombinant protein. Confirm that it has not expired, has not undergone repeated free-thaw cycles (>2 cycles), and it is freshly added to the culture medium. Ensure that the final concentration of GM-CSF in the DC medium is within the recommended range (typically 10–25 ng/mL).

Third, minimize mechanical disturbance during culture. Handle petri dishes gently when removing them from or returning them to the incubator, as developing dendritic cell clusters are loosely adherent.

If reagent quality and handling are appropriate, supplement cultures once more with fresh DC medium containing murine GM-CSF and extend incubation for an additional 2 to 3 days to evaluate cell colony formation.

### Problem 3

Low percentage of CD11c^+^ cells after differentiation (step 13).

### Potential solution

Under standard GM-CSF-based differentiation conditions, BMDC cultures typically yield 70-90% CD11c^+^ cells.^4^^,^^9^

In our experience:

Use cultures with >70% CD11c^+^ cells for downstream co-culture or functional assays without substantial impact on experimental readouts.

Cultures with 50–70% CD11c^+^ cells may still be suitable for dendritic cell maturation or antigen presentation assays, however, interpret functional outcomes cautiously.

If CD11c^+^ cell purity falls below 50%, repeat bone marrow isolation and differentiation. To improve CD11c^+^ cell yield, ensure the use of non-TC-treated petri dishes and avoid excessive disturbance of loosely adherent cells.

### Problem 4

The purity of CD8^+^ T cells after magnetic isolation is low (step 23).

### Potential solution

Do not exceed the recommended cell number per LS column during magnetic separation. Make sure to scale up the reagent volumes appropriately according to total cell amount. Perform the labeling steps at 2–8 °C to maintain efficiency.

## Resource availability

### Lead contact

Further information and requests for resources and reagents should be directed to and will be fulfilled by the lead contact, [Olivia M. Merkel] (olivia.merkel@lmu.de).

### Technical contact

Technical questions on executing this protocol should be directed to and will be answered by the technical contact, [Min Jiang] (min.jiang@cup.uni-muenchen.de).

### Materials availability

This protocol did not generate unique materials or reagents.

### Data and code availability

This protocol did not generate datasets or code.

## Acknowledgments

This project was partially funded by the 10.13039/501100002745Bavarian Research Foundation (AZ-1449-20C) and ERC-2022-COG-101088587. M.J. acknowledges financial support from the 10.13039/501100004543China Scholarship Council. The graphical abstract was created using Biorender.com.

## Author contributions

Methodology, M.J., D.M., and A.N.; writing – original draft, M.J.; writing – review and editing, M.J. and O.M.M.; funding acquisition, resources, and supervision, O.M.M.

## Declaration of interests

The authors declare no competing interests.
